# Factors influencing attendance in a structured physical activity program for Aboriginal and Torres Strait Islander women in an urban setting: a mixed methods process evaluation

**DOI:** 10.1186/1475-9276-12-11

**Published:** 2013-01-24

**Authors:** Karla J Canuto, Belinda Spagnoletti, Robyn A McDermott, Margaret Cargo

**Affiliations:** 1 Sansom Institute for Health Research, University of South Australia, Adelaide, Australia

**Keywords:** Aboriginal, Torres Strait Islander, Physical activity, Women, Lifestyle program, Health promotion, Barriers, Facilitators, Participation

## Abstract

**Background:**

Aboriginal and Torres Strait Islander women experience higher rates of obesity, chronic disease, and are less active than non-Indigenous Australian women. Lifestyle programs designed to increase physical activity and encourage healthy eating are needed to ameliorate this disparity. The aim of this study was to identify participants’ perceived barriers and enablers to attend group exercise classes as part of a 12-week fitness program.

**Methods:**

To understand the factors that influence attendance, a mixed method process evaluation was undertaken in which a quantitative measure of attendance in the group exercise classes was used to identify cases for further qualitative investigation. Aboriginal and/or Torres Strait Islander women aged 18 to 64 years were recruited to a research trial of a fitness program. The 12-week program included two 60-minute group exercise classes per week, and four nutrition education workshops. Semi-structured interviews were conducted at program completion. Participants were stratified by attendance, and interviews from the highest and lowest 25 percentiles analysed. Rigour was strengthened through use of multiple data analysts, member checking and prolonged engagement in the field.

**Results:**

Analyses of the post-program interviews revealed that participants enrolled in the program primarily for the perceived health benefits and all (with one exception) found the program met their needs and expectations. The atmosphere of classes was positive and comfortable and they reported developing good relationships with their fellow participants and program staff. Low attendees described more barriers to attendance, such as illness and competing work and family obligations, and were more likely to report logistical issues, such as inconvenient venue or class times.

**Conclusions:**

Attendance to the ‘Aboriginal and Torres Strait Islander Women’s Fitness Program’ was primarily influenced by the participant’s personal health, logistics and competing obligations. Low attendees reported more barriers during the 12-week period and identified fewer enabling factors than high attendees.

**Trial registration:**

Australian New Zealand Clinical Trials Registry ACTRN12610000224022

## Background

Compared to non-Indigenous Australian women, Aboriginal and Torres Strait Islander women are more likely to be obese, have lower levels of physical activity and higher rates of type 2 diabetes mellitus (T2DM) [1]. It is currently estimated that the life expectancy gap between Indigenous and non-Indigenous Australian women is ten years [2].

There is conclusive evidence that regular physical activity is an effective primary and secondary preventive measure against chronic diseases such as T2DM [3], and clinical trials have shown that the combination of physical activity and nutritional advice can reduce weight [4], blood pressure [5,6], incidence of T2DM [7], and improve glycemic control in diabetics and reduce other diabetes related complications [8,9].

Several lifestyle programs to prevent and/or manage T2DM among Indigenous adults, or behavioural risk factors for the development of chronic disease in Indigenous populations have been implemented [10-24]. Most programs have found some short-term health improvements such as increased physical activity rates, improved nutrition and small decreases in weight or waist circumference. Reviews of these, however, point to the poor overall quality of evaluation research [15,25]. These studies, for example, are often not accompanied by process evaluations. Process evaluations are important for providing insight into how programs are implemented and whether difficulties are encountered in the field [15].

Implementation assessment has been long recognised as a critical element of program evaluation, as it can strengthen the basis for understanding differences in results and explaining how breakdowns in implementation processes can impact hypothesised causal pathways [26-28]. As such, process evaluations strengthen the internal validity of program effectiveness evaluations. In a comprehensive review of process evaluations in public health Linnan and Steckler contend that, as a minimum, evaluations should collect data on contextual factors that influence program implementation, including the recruitment of participants and reach, or attendance [15].

Indigenous people across a number of health programs have reportedly low attendance rates. Clifford et al. completed a review on lifestyle interventions that targeted Indigenous Australians. Of the four included physical activity and nutrition programs, all reported less than 80 per cent of participants completing the study [15]. Attendance information is essential for interpreting the outcomes of effectiveness evaluations and for designing programs that address the factors that impede attendance. Even the best designed program will fail to achieve the desired outcomes if participants do not attend.

The ‘Aboriginal and Torres Strait Islander Women’s Fitness Program’ was a structured, progressive 12-week group program aimed at reducing waist circumference and improving metabolic health. The program is described in further detail elsewhere [29]. Logistical issues such as transport, childcare and financial constraints were identified by the specially formed local advisory committee as potential barriers to attendance; consequently the program was delivered with no cost to participants and offered transportation and crèche. The evaluation of the anthropometric and metabolic measures found only a modest impact of the program on the active group compared to the waitlisted participants [30] however the participation rates were low. This objective process evaluation explores some of the issues affecting exercise class attendance.

The study aim was to identify the perceived barriers and facilitators to attendance of Aboriginal and Torres Strait Islander women registered in a 12-week structured physical activity and nutrition program.

## Methods

### Study sample and data collection

Aboriginal and Torres Strait Islander women aged 18 to 64 years residing in Adelaide, South Australia, were invited to participate in the research project. The inclusion criteria is described elsewhere [29]. Participants were recruited in three intakes over a period of approximately one year. Each intake was randomised to form two groups; the ‘active’ participants who were assigned to start the 12-week program shortly after their baseline assessment and the ‘waitlisted’ participants who had a 12-month delay before commencing the program. The active group from the first and second intakes, groups ‘A’ and ‘C’ had group sessions (exercise classes and nutrition workshops) in the central business district (CBD) of Adelaide, while the third intake, group ‘E’ had group sessions in a suburb approximately 30 kilometres from the Adelaide CBD.

### Research design

To understand the factors that influence attendance, a mixed method process evaluation was undertaken in which a quantitative measure of attendance in the group exercise classes was used to identify cases for further qualitative investigation. High and low attendees were identified objectively from attendance records across the 12-week program. These cases were used to understand the factors underpinning variation in attendance [31].

The study was informed by Chen’s program planning framework which identifies program and participant-related factors as influencing aspects related to program implementation [26]. Of interest to this study was attendance, as a key indicator of program implementation. Programmatic factors were initially conceptualised as they related to the implementers (e.g., exercise instructors), implementing system (e.g., organisational aspects of the program), and characteristics of the structured physical activity program. Prevailing social-ecological perspectives applied to Indigenous health [32,33] guided the conceptualisation of participant-related factors influencing engagement. Here, the barriers and facilitators to attendance were conceptualised as emanating from the participant (i.e., individual), their family, and their non-familial interpersonal networks.

### Participation in exercise class

Each participant was encouraged to attend two classes per week during the 12-week program. The days and times of the classes were organised around the preferences of each group. A staff member recorded participant attendance to classes. Attendance was noted as a participant being present for the session. There was no measure of effort or exertion. A continuous measure of participation was computed and constituted the quantitative component of the mixed method process evaluation [34].

For these analyses participants were purposively sampled based on their attendance to exercise classes. Attendance was stratified and only participants located in the top 25th percentile and bottom 25th percentile were analysed. The bottom 25th percentile included eight participants who had attended eight or less classes, these are referred to as the ‘low attendees’. The top 25th percentile, ‘high attendees’, included eight participants, each of whom had attended 18 or more classes. These two extreme groups were chosen for analysis to compare and their perceived barriers and enablers to attendance to the exercise sessions.

### Post-program interview

Participants were invited to take part in a one-on-one semi-structured interview following completion of the 12-week program. The interviewer was a local Aboriginal woman with extensive experience in conducting research interviews. Interviews took place in workplaces, homes, at the university and, if a face-to-face interview was not possible, over the telephone.

The interview guide was designed to capture individual, interpersonal and environmental factors influencing participant engagement in the program in relation to their family, work, study and community responsibilities and obligations. The interviewer used exploratory, amplificatory and explanatory probes to clarify and better understand the individual, family, social and work-related factors that acted as barriers or facilitators to attendance.

Interviews were digitally recorded and professionally transcribed. Following each interview, the interviewer completed a contact summary sheet detailing the quality of responses, the main issues or themes that emerged and any changes in body language throughout the interview [35]. The contact summary sheets were used to contextualise the interview, for instance, interpreting the interview in relation to the willingness of the participant to share their story.

### Demographic characteristics of participants

Demographic data was collected at baseline via questionnaire. This questionnaire captured personal information and socio-demographic information, including home address, date of birth, employment status, and highest educational attainment. Household information was also included, such as household income and the number of adults and children in the home. Even though all forms were received, not all questions were completed.

### Health assessments

Objective assessments of metabolic health were taken including; basic anthropometrics (height, weight, waist and hip circumference) and blood pathology (fasting blood glucose, insulin, lipids, and glycated haemoglobin (HbA1C)). Further details of these assessment protocols, including the equipment and assays used, are described elsewhere [29]. Assessments were conducted at multiple time points, however, only the baseline assessments (before the 12-week program) are presented here.

### Data analysis

The demographic and metabolic health data of the high and low attendees were compared using mean values for continuous data and numbers of occurrence for categorical variables. Means were calculated using Stata 11 [36].

To ensure that interview data were coded in the context of each case, audio files were listened to, the transcripts read and re-read. Each interview was analysed independently by two researchers. Thematic network analysis was applied to identify basic themes, organising themes and higher-order global themes [37]. Segments of interest within the transcript, or ‘meaning units’ were identified and assigned a basic code to reflect the type of barrier or facilitator to participation [37]. Basic codes (e.g., death in the family, personal injury) were compared and contrasted and assigned into conceptually meaningful basic themes (e.g., logistics, instrumental support). Following this, other themes were created that represented internally homogeneous and externally heterogeneous groups of meaning units [38]. Guided by the Chen’s program planning framework [26] and existing social-ecological frameworks adapted for Indigenous populations [32,33], these organising themes were linked to three high-order global themes to capture the factors related to recruitment and attendance: program motivation, personal circumstances and program experiences. Qualitative data were analysed using NVivo Version 9 [39]. Credibility of the findings was strengthened by the analytic triangulation of two researchers coding the complete set of interviews. Regular peer debriefing with a third researcher was also important for posing rival interpretations of the data.

## Results

### Baseline demographic and metabolic health of high and low attendees

The socio-demographic factors and baseline health assessments of the low and high attendees were compared. Low attendees were more likely to be employed (7 low attendees compared to 5 high attendees), were less likely to have dependants under the age of 18 (4 low attendees compared to 2 high attendees), and on average lived 2.6 km further from where classes were held. Compared to high attendees, low attendees were on average nearly 4 years younger and 7.6 kg heavier, with a waist circumferences 9.5 cm larger, and a fasting insulin 5.6 mmol/L higher. There was no difference between groups for educational attainment, annual household income, fasting glucose, HbA1c or lipid profile. These differences were noted, but due to the small number of participants no statistical analysis was conducted and no firm conclusions can be drawn about their significance.

### Participant attendance at exercise classes

On average, attendance at the group exercise classes was low. Participants were encouraged to attend a total of 24 classes; two classes per week for twelve weeks; although group A was offered 5 classes a week to meet the needs of the participants. Of the 51 participants the average number of classes attended was 9.5 (95 % CI, 7.4 - 11.6). Seven participants did not attend any exercise classes, six of whom withdrew from the program. Of the participants who completed post-program interviews, the eight highest attendees attended an average of 20.4 classes (range 18 – 30 classes) and the eight lowest attendees attended an average of 6.5 classes (range 2 – 8 classes).

### Participant interviews

Interview length averaged 40 minutes. Interview quality was reported by the interviewer providing an overall subjective rating based on the richness of responses, the openness of the participant to share their story and any issues that arose (e.g., background noise, interruptions). There was little difference in interview quality between the high and low attendees overall. The atmosphere created by the interviewer contributed to most of the women feeling comfortable to speak freely and share their story.

### Factors influencing attendance

**Figure 1:**
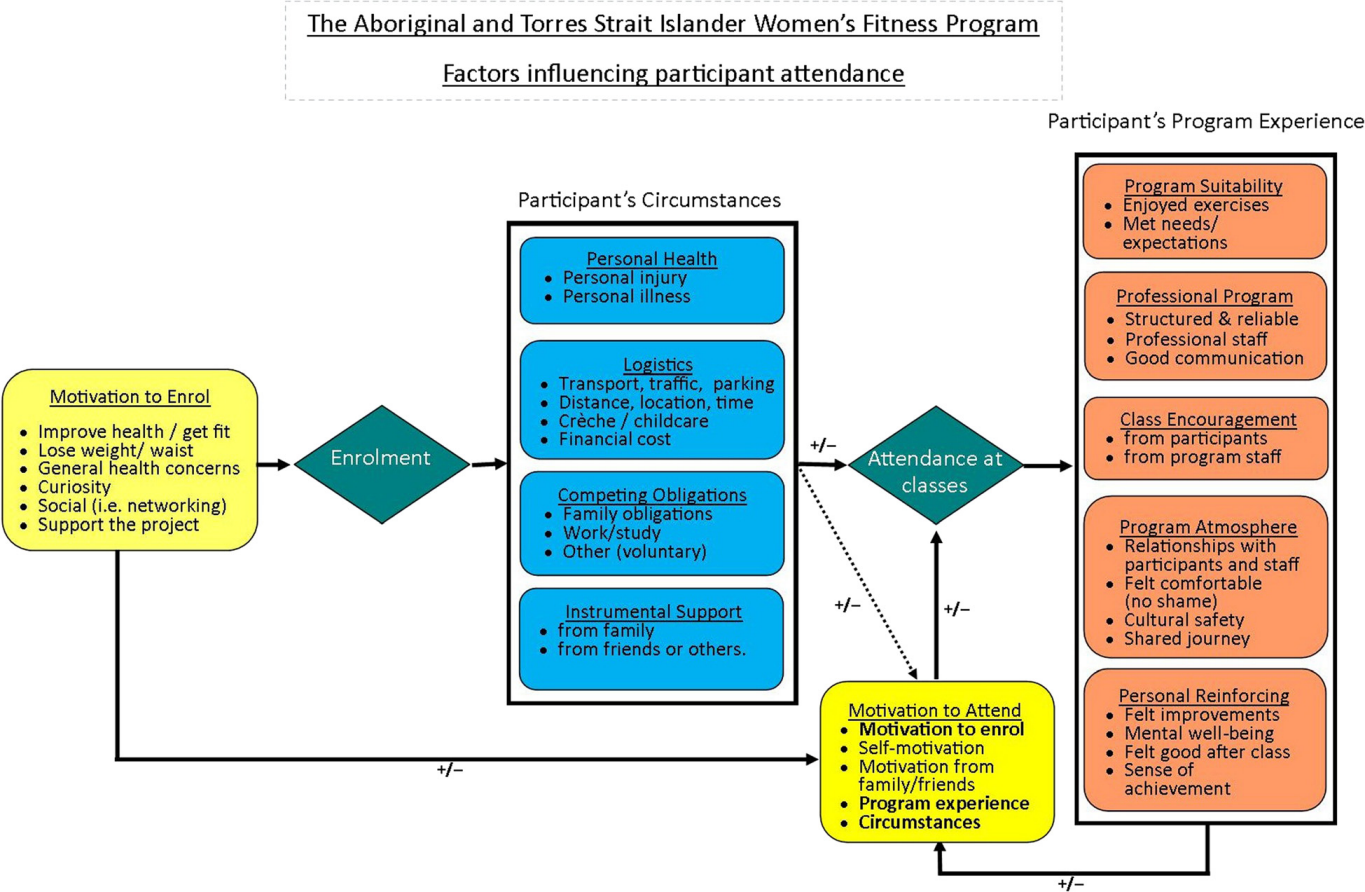
Factors influencing participation in the structured exercise classes.

The factors that influenced class attendance are synthesised and displayed in Figure 1. Motivations primarily related to personal health led participants to enrol in the program. Class attendance was influenced by participant’s circumstances and depending on the participant; these circumstances had mitigating effects, and enabled attendance, or acted as barriers to class attendance. For some participants barriers were modified by the availability of instrumental support, usually provided by a family member or friend. Once a participant attended the program, their experience influenced their continued motivation to attend the classes. Initial motivation to enrol also contributed to their ongoing attendance motivation, as did their logistical issues, competing obligations, and the instrumental support they received from those outside the program. Factors that had a positive or negative influence have symbols beside the arrows,‘+’ and ‘-’, respectively. Each key factor influencing attendance is described in detail below. Participant quotations are used to enhance the interpretive validity of the findings.

### Program motivation

The global theme of program motivation was comprised of participant’s initial motivation to enrol, and their motivation to attend the fitness program following enrolment. As depicted in Figure 1, motivation was not static, but changed as women gained experience with the program.

#### Motivation to enrol

The motivations driving participants to enrol were similar regardless of attendance level. All participants, with the exception of one, identified a desire to improve their personal health and wellbeing in general or to mitigate an existing health issue as the impetus for registering. For example, *“I just thought it might be a good way to kick start my health again.”* For others, their health concern was linked to improving a specific health-related outcome, for example, *“to hopefully get fit and lose a bit of weight”* or improve an existing medical condition, *“I’ve got high blood pressure and I want to try and get off as much tablets as I can.”* Only one participant did not mention personal health as a motivation for enrolling, however she was a low attendee who provided a short phone interview which had gaps in the transcript due to telephone line interference and background noise.

Some participants were curious about the program and thought they would see what it had to offer, *“I just thought I'd just try it and see what it was like”* and because there was no financial cost they felt they had nothing to lose, “*any exercise you can do for free, why not!*” Other participants were attracted by its social aspect to either *“meet new people”,* to network with other Aboriginal and Torres Strait Islander women, or thought it was a great opportunity to *“get together as a group of Aboriginal and Torres Strait Islander people …and do it.”* Finally, a secondary motive for joining the program related to participants supporting the research, *“…and also to support the program because I think it’s a really good program.”*

#### Motivation to attend

From the women’s stories we identified five sources of motivation related to their ongoing attendance, specifically, their original motivation to enrol in the program, their self-motivation to be active, the motivation they received from family and friends, motivation gained from experience they had in the program, and their personal circumstances.

Self-motivation was realised through participants prioritising their needs and/or a strong commitment to completing the program. *“I just made that a priority for me and then I just worked around that.”* Participants were also motivated and encouraged to attend regularly by their family and friends:

“… my family and friends – they knew I was participating in the program and they thought it was really good and especially my Mum – she reminded – “Oh, you’ve got that thing tonight at six – remember that.”

Overall, a participant’s program experience seemed to have either a positive or a negative impact on their motivation for ongoing attendance. *“I loved it; I enjoyed going there, that’s why I went there all the time.”* When asked if there was anything about the program they found particularly motivating, a participant explained:

“I think just the atmosphere of everyone was willing to give it a go, that was motivating and the fact that they were also supportive, just (staff members) – they were like really supporting. They were like “C’mon …, you can do another one of those.” And plus all the ladies – I found them really supporting because I was the youngest one and they were all just encouraging me little by little you know. So it was just the atmosphere really that was motivating me to keep going.”

The personal circumstances of each participant also impacted on their motivation to attend classes. Some of these factors are described below and even though most were not specifically linked by some participants to their motivation others noted that factors such as logistical issues could make them feel ‘reluctant’ to attend.

### Participant circumstances

A participant's personal circumstances influenced their ability to engage in the program regardless of their motivation to enrol. Personal health issues, ‘logistics’, competing priorities, and instrumental support acted to either constrain or enable their participation.

#### Personal health

Personal health issues surfaced as a strong barrier to class attendance. Low attendees were more likely to report long-term injuries or illnesses during the 12-week program:

“Well for me I know that I missed the first couple of weeks because I was actually injured from netball.”

High attendees, on the other hand, only experienced mild or short-term illnesses or injuries.

#### Logistics

For high attendees logistical factors were not identified as barriers to attendance although, for some, parking was noted as a nuisance. Most high attendees found the class times and location convenient, as illustrated in the examples below:

“I found it good, like the location, because it was in close proximity to my work”,

“The timing was really good because they do it at 6-7[pm] … Tuesday and a Thursday so yeah if it was any earlier then maybe I wouldn’t have been able to.”

Whereas, for some low attendees, the location was a barrier to attendance,

“Yeah I did half the program. I stopped because it got too far away for me.

Even though some logistical issues were more ‘inconveniences’ rather than barriers, participants noted the effect on their motivation to attend:

“Sometimes it made me reluctant. Do I feel like sitting through traffic?”

Crèche was often mentioned by the women who used the service; however, it is unknown if its absence would have been a limiting factor for these participants or just an added convenience. The fact that the program was ‘free’ was regularly mentioned by interviewees. Some participants also discussed the cost of gyms and other activities:

“*…a lot of people can't afford something like that (gym membership) because most people in (suburb) are on the dole or on a pension (government welfare).”*

#### Competing obligations

Competing obligations were barriers to class attendance and included family obligations, work, study and/or other commitments. The impact of competing obligations varied significantly; some were one-off events, such as a family member’s birthday, while others were short-term priorities, such as being asked to work back late for several days, or study commitments, *“going away on block study caused a bit of an interruption.”* High attendees reported less competing obligations, while low attendees either reported having several obligations, or they experienced major life events that completely hindered attendance for a significant block of time, such as death in the family.

“I was able to keep up with it for the first month, then after the first six weeks I started decreasing because I had other (health and family) issues and other (study and work) interruptions.”

“…but then unfortunately during the last few weeks I had a death in the family so I had to go out of town so that didn’t really help because I was out – I was completely out in the middle of nowhere.”

#### Instrumental support

Instrumental supports emerged as tangible or practical supports normally offered by family members or friends. In our analysis, we found that instrumental supports modified a competing obligation or logistical issue. For example a participant who was normally responsible for getting her children ready in the morning and taking them to childcare and kindergarten received instrumental support from her husband so that she could attend morning exercise classes:

“So obviously my husband is quite supportive in terms of like getting the kids up … because I would leave home by 6:30 am.”

Another participant who attended evening classes was assisted by her mother who loaned her a car, *“I used her (mother’s) car for most of the time … so I wouldn’t be catching the buses that late.”* However, having support did not prove essential to attendance. Participants who did not identify any barriers or competing priorities did not require support to enable their attendance.

“Well I didn’t have really, any family commitments, because my daughter’s away, and really, not that much work really, it’s just your 9 to 5 during the week, so I didn’t have really, no problem about attending any sessions or exercise classes and things like that, so, no that was pretty easy for me.”

### Participant’s program experience

All participants, with the exception of one, reported their program experiences to be all positive. The program experience reflected the program suitability, professionalism of the program, class encouragement (from participants and staff), program atmosphere, and personal reinforcing factors.

#### Program suitability

Participants reported that the program was a good fit for themselves and other participants. It met their needs and expectations. They particularly liked the fact that the activities accommodated participant’s different fitness levels:

“(they) catered for all different levels, you know what I mean, like say if you were just a beginner or something … you could work at that level or you could be higher fitness level, whatever and it's a, it accommodates to that as well.”

Participants enjoyed the exercises, and this impacted their motivation to attend, *“I was really enjoying the classes so that just kept me coming back for more.”*

Unfortunately one participant, a low attendee, found that the program was not what she expected. She also felt the program was not suited to her fitness level, consequently she felt “uncomfortable.”

“Well at first I thought the circuit class was going to be good but I found some of the exercise was a little bit hard for me … so yes I did an adaptation of some of those exercises that the instructor would say if you can’t do that one well you do it this way and she’d show you a different way and that was good but I still felt a bit uncomfortable … maybe if it was like one group was over one side of the room and the other group was on the other side so that we were able to go at a slower pace…”

#### Professional program

The professionalism of the program contributed to the overall program experience. Most women reported that they felt confident that the staff were knowledgeable and professional, reliable and had good communication skills. They also found the program well organised which made the program *“easy and not a chore.”*

“I thought that it was really good and run really well, kept up to date with everything and good communication, people were friendly.”

#### Class encouragement

During the interviews many women discussed the encouragement they received from program staff and their fellow participants. Participants indicated that they found the encouragement during class motivated them to put their full efforts into the activities, thereby influencing their motivation to attend the classes.

“When the instructors sort of pushed you, and challenged you to try the new things actually it was surprising to think, oh my goodness I can actually do that exercise…”

“I think that was just the environment and the people that were there, and a lot goes to the staff that were doing it. Like you know they were very encouraging whether it was (staff member) or (researcher) or any of the instructors that were there, everyone was encouraging and yeah.”

#### Program atmosphere

The atmosphere of classes had a significant impact on the participants’ program experience. This included the relationships that the women formed with fellow participants and program staff. When participants were asked what they found particularly motivating about the class most talked about the group environment, a positive atmosphere and feeling comfortable. All participants made reference to liking the fact that the program was “*a group of women and they were going to be with other Nungas or other Aboriginal, Torres Strait Islander women”* or that the participants shared goals or were on a similar journey or that they *“felt comfortable.”*

‘I think it was supportive like everyone was really encouraging … being all Aboriginal women was good, you didn’t feel out of place … It was a program that specialised to make their environment as comfortable as it could be for participation … it just helped with the participation or your ability to do some of the exercises.’

There was a sense of women sharing a journey together and in a culturally safe environment in which they felt ‘no shame’. These factors contributed to their program experience and ongoing motivation to attend the classes.

#### Personal reinforcing experiences

All participants reported perceived improvements on one or more aspects of their physical or emotional well-being. The most common reinforcing factors were participants perceiving physical improvements such as feeling the exercises becoming ‘easier’ or feeling ‘fitter’ during the 12-week program.

“I could feel the difference in the fitness level of myself. I guess and I was able to do some of the exercises a lot more easier than when I first started.”

These physical improvements extended to women talking about losing weight, sleeping better, having improved blood pressure, as well as experiencing reduced pain in their joints and improved mobility.

The women also reported having ‘more energy’ *“…like I said, I feel fitter, healthier, more energy”,*

Beyond improvements in energy, women also shared stories about perceived improvements to their mental well-being, such as feeling less stressed or feeling better about themselves.

“…since I started the program and exercising and stuff it just lifted me and helped me to feel better about myself. I wasn’t depressed or anything, but I just didn’t have any motivation to do anything, whereas now I know with exercise and eating well you feel good and you know, it’s a benefit.”

Participants also commonly reported that they *‘felt good’* after the exercise sessions. Additionally high attendees reported a sense of achievement or accomplishment:

“…it was hard work, and we felt really good after it, a real high after it. And it took away all that stress and you felt really happy.”

As discussed earlier and depicted in Figure 1 the participant’s circumstances and their program experience fed into their motivation to attend the classes, which was also influenced by their initial motivation to enrol, their self-motivation and the motivation that they received from their family and friends.

## Discussion

This is one of the first mixed method process evaluations embedded with a pragmatic controlled fitness trials in an Indigenous population to examine the barriers to participation of high and low attendees. Despite sharing positive motivations and positive program experiences with high attendees, those who struggled to attend classes either experienced several competing obligations and logistical issues that made attending very difficult in the absence of instrumental support, or they had a major event occur, such as a death in the family. In particular, low attendees experienced more competing family obligations and work obligations.

There were similarities in the stories shared by the women in the positivity of their program experience and program motivations. The motivations of the women to enrol in the program, and to continue attending the program, were also similar and there did not appear to be a discrepancy between the two groups. Participants reported enrolling in the program primarily for the perceived health benefits. Participants perceived the program to be of high quality and all but one only had positive things to say about their program experience. Participants felt comfortable and reported a positive program atmosphere. The fact that the program was designed by an Indigenous woman, for Indigenous women, and was implemented in a culturally safe environment contributed to the women’s positive program experience.

A review of the literature did not reveal any qualitative studies on the barriers to participation of Indigenous women engaged in equivalent structured group-based lifestyle intervention. However, previous studies on at-risk minority populations report similar findings. The community health assessment and promotion project (CHAPP) was a ten-week group-based exercise and nutrition intervention in an urban setting, which provided crèche and transportation [40]. All participants were obese Black-American women. CHAPP staff followed up non-attendees and documented their self-reported reasons for non-attendance. Similar to our study, the most common reasons related to competing obligations (family and work) and personal illness [40]. Likewise, a centre-based program for physical activity among low-income, Black-American families with young children found that competing obligations (work and school) were the primary reason for non-attendance [41].

In another study, focus groups amongst African-American women on the perceptions of physical activity and personal barriers and enablers found that lack of time, lack of self-motivation and competing priorities (putting their family first) as the most frequently cited perceived barriers [42]. A qualitative inquiry into the barriers and potential strategies for promoting physical activity among urban Indigenous Australians using focus groups also found that competing family obligations (such as raising children) was a barrier to being active [43]. Participants also identified personal illness and injury, the perceived financial cost and the lack of sustainable programs as barriers [43].

The role of competing family obligations cannot be under-estimated. With 40 per cent of Aboriginal and Torres Strait Islander Australians under the age of 15 and with a higher number of children per household, compared with non-Indigenous women [44], Aboriginal and Torres Strait Islander women have considerable family responsibilities, not to mention cultural obligations. It is of interest to note, however, that low attendees had fewer dependents under the age of 18 than high attendees. Our interviews suggest that the presence of instrumental support for high attendees and absence of such support for low attendees can be attributed to non-attendance in the face of logistical issues and competing obligations. Like these other studies, the program was implemented in an urban setting, and some of the women were commuting between home and work. These factors may be less important in rural and remote settings where travel and parking may be more convenient and instrumental supports may be closer to home.

An older, comprehensive, review of physical activity determinants found that self-motivation, support from a spouse and reinforcement from program staff and or activity partner influenced adherence [45]. Self-motivation was a stronger enabler for high attendees than it was for low attendees, and the presence of instrumental support was a key factor in offsetting participant’s competing obligations at home and work. They also found that perceived convenience or distance to travel can predict participation as can a perceived lack of time. Similar to our study, they noted that an interruption in usual routine, such as a major life event, can interrupt or discontinue participation even in the most motivated and well-intentioned participant [45]. Dishman et al. also found that strategies that effect change, all have a common dimension of social reinforcement and appear more successful when conducted in groups [45].

### Researchers’ lens

The collective process of data analysis and interpretation challenged the researchers to reflect critically on their personal biases and how these biases were reflected in their interpretations of the data [38].The first author is a Torres Strait Islander woman with strong connections to family and community and expertise in exercise physiology and in the design and management of health promotion programs. Even though she is not originally from the community where the program was implemented, her life and work experiences contributed to the cultural understanding and insight used to interpret the women’s stories. As the designer and evaluator of the program, she attended most exercise classes, was abreast of the day-to-day running of the project and all communications with participants. During the course of the project she built strong relationships with most of the participants and came to know the circumstances affecting their lives. This knowledge deepened her insights into the data.

### Implications

The findings of this study have implications for the design and delivery of group-based exercise programs targeting Indigenous women living in urban areas. Based on our findings, there may be some benefit to implementing pre-program workshops with participants to identify potential barriers and solutions to their program attendance. We found, for example, that it was important to provide instrumental supports like crèche and transport to enable some women to attend the exercise classes. Having a process that identifies and addresses the barriers emerged is important. It is additionally important that the program invest resources to develop a professional program that offers consistent training, technical support and feedback to staff responsible for implementing the program. Offering a well-organised program, run by qualified, supportive and motivating staff contributes to a positive and engaging atmosphere for participants. We also found that running the program only for Aboriginal and Torres Strait women, in a non-gym setting, created a comfortable place for women to exercise and positively influenced their motivation to attend.

### Limitations

The study findings should be interpreted in light of the following two limitations. First, the study is based on a small number of interviews from participants who completed the program and consented to an interview. Interviews from participants who withdrew from the program or were lost to follow-up would likely have provided greater insight into the factors influencing participation. Consequently, the circumstances and barriers affecting the participants that were not interviewed are unknown. Second, although the semi-structured interview was designed to capture participant’s barriers and enablers to attendance in relation to their life circumstances, how they came to enrol in the program and what motivated them to continue attending, the interview guide probed into other issues as well, and this may have detracted from the richness of the women’s stories about their engagement.

## Conclusions

This mixed method study uncovered some key similarities and difference**s** between high and low attendees of the Aboriginal and Torres Strait Islander Women’s Fitness Program, giving some insight into the factors that influenced their attendance. There were five key sources of motivation to attend classes; their motivation to enrol, their self-motivation for exercise, motivation from family and friends, their program experience and their circumstances. Most women talked extensively about their experience with the program, including the relationships they developed with the staff and other participants, enjoying the classes and the exercises, feeling safe and ‘comfortable’ and perceived improvements in their own health and well-being. High and low attendees differed in their circumstances during the program with low attendees experiencing more barriers, particularly logistics and competing obligations in the absence of instrumental support, and fewer enabling factors. Future programs should focus on reducing barriers to attendance. Barriers that are unavoidable and unpredictable will always be present; however, some can be modified by instrumental support. Pre-program workshops could prove useful to help identify potential instrumental supports that service providers could offer and/or assist participants to identify sources of instrumental support from their family and friends.

## Ethics

The project gained ethics approval from the University of South Australia’s Human Research Ethics Committee and the Aboriginal Health Council of South Australia’s Human Research Ethics Committee.

## Community consultation

The project sought the guidance, advice and support of their collaborating partners, Nunkuwarrin Yunti of South Australia Inc. and the Aboriginal Sobriety Group Inc. in Adelaide and their specially formed advisory committee of local women.

## Competing interests

The authors declare that they have no competing interests.

## Authors' contributions

KJC developed the 12-week program, contributed to the study design and was involved in the data collection and delivery of the project. RAM developed the study design and led the NHMRC Project Grant. BS and KJC coded and conducted the analysis under the supervision of MC. BS was also involved in the data collection and delivery of the project. MC also contributed to the study design. KJC was primarily responsible for drafting the manuscript. All authors approved the final manuscript.
